# Closing the health equity gap: A role for implementation science?

**DOI:** 10.1371/journal.pmed.1003762

**Published:** 2021-09-14

**Authors:** Beryne Odeny

**Affiliations:** PLOS Medicine, San Francisco, California, United States of America

## Abstract

Beryne Odeny discusses strategies to improve equity in health care and health research.

WHO defines health equity as “the absence of unfair and avoidable or remediable differences in health among population groups defined socially, economically, demographically, or geographically or by other means of stratification” [1]. Yet, contrary to this fundamental aspiration and the international mandate on universal health coverage (UHC), almost 50% of the world’s population does not receive needed health services, and progress toward health equity remains elusive [2].

## Equity in clinical research

In a new study published in *PLOS Medicine*, Muthukumar and colleagues address underrepresentation of minority populations, a pressing equity issue in clinical research. In a systematic analysis of ClinicalTrials.gov, the authors investigated the frequency of English language requirement in interventional United States (US) clinical trial eligibility criteria [3]. Of 14,367 trials registered from January 2019 to December 2020, approximately one-fifth specified language requirements. They found that approximately 19% (*n =* 2,727) of clinical trials required English language proficiency, while approximately 3% (*n* = 390) had accommodations for non-English language. Trials related to Coronavirus Disease 2019 (COVID-19) and other infectious disease were less stringent, while depression-related trials were most stringent. That language barriers limit access to the benefits of participation in research targeting depression and noncommunicable diseases (NCDs), which disproportionately affect marginalized groups, is disconcerting. This goes against the grain of the Belmont Report on justice and equality in research conduct and propagates disparities in understanding effectiveness, appropriateness, and acceptability of interventions in marginalized groups [4].

## Equity in healthcare

Undertreatment or underprescription among distinct groups of individuals leads to inequities in translation of evidence to healthcare practice. This has been investigated in NCD care models as demonstrated by a recent population-based cohort study by Eastwood and colleagues. The study revealed that people with type 2 diabetes from African, African Caribbean, and South Asian backgrounds are less likely to be prescribed statins than people of White European ethnicity in the United Kingdom [5]. Statins lower cholesterol and are a crucial for mitigating the risk of atherosclerotic and cardiovascular events (ASCVD), including heart attack and strokes, in people with type 2 diabetes. People of African/African Caribbean and South Asian ethnicity were 24% and 9% less likely, respectively, to receive statins than people of European ethnicity. Among patients receiving statin therapy, the median times to initiation were 79, 109, and 84 days for people of European, South Asian, and African or African Caribbean ethnicity, respectively. The authors estimated that up to 13,000 ASCVD-related deaths could be avoided by achieving equality in statin prescription in people with diabetes across the ethnic groups. These findings serve as a stark reminder of the persistence of inequities that are life-threatening for marginalized groups.

## Impact of inequity on health outcomes

Disproportionate burdens of poor health outcomes on vulnerable communities in the wake of the COVID-19 pandemic have exacerbated health inequities. In the US alone, racial and socioeconomic inequities, established drivers of health inequity, have a demonstrable link to disparate COVID-19–related health outcomes, from testing rates to mortality [6]. The COVID-19 pandemic has laid bare the cost of health inequities—a cost that is heavily borne by the most vulnerable. In a cross-sectional study, Stokes and colleagues assessed National Center for Health Statistics (NCHS) data on direct COVID-19 and all-cause mortality in 2,096 US counties from January 1 to December 31, 2020 [7]. They investigated the excess deaths not assigned to COVID-19 and examined variations in excess deaths by counties. They found that “for every 100 deaths directly assigned to COVID-19 in official statistics, an additional 20 deaths occurred that were not counted as direct COVID-19 deaths.” The proportion of excess deaths was higher in counties with lower average socioeconomic status as it was in counties with more non-Hispanic Black residents. The authors conclude that health equity must be prioritized in the policy response to the pandemic, to prevent escalation of inequities.

## A role for implementation science

In the quest to redress the health inequities present at multiple societal levels as illustrated by these examples, implementation science (IS) has the potential to sharpen strategies to achieve health equity. So as to break the cycle of inequity, it is crucial to (1) systematically parse and understand the layers of inequity; (2) identify strategies to promote equity; and (3) generate metrics for quantifying and monitoring disparities so as to guide progress toward eliminating them [8,9].

IS provides a structure for analyzing and understanding context to identify strategies to enhance adoption of interventions in marginalized groups, to foster health equity, drawing on multidisciplinary tools to interrogate contextual layers: patient, provider, health systems, sociocultural, and policy layers. These tools include theoretical frameworks, compilations of implementation strategies, implementation outcomes, and pragmatic research designs. Comprehensive frameworks such as the Consolidated Framework for Implementation Research (CFIR) and Reach Effectiveness Adoption Implementation Maintenance (RE-AIM) and Health equity implementation framework [8,10,11] can be used to define the equity gap, understand why researchers exclude certain groups and, conversely, why some marginalized groups avoid clinical research, and measure diversity in clinical trials. They can also aid our understanding of healthcare access, effectiveness of interventions, and outcomes in marginalized groups.

## Implementation strategies

Evidence-based implementation strategies can also be used to promote uptake of interventions in distinct groups to promote equity. Examples of strategies include education, reminders, audits and feedback, shared decision-making, demand creation, continuous quality improvement, and decentralization of services [12]. Education targeting researchers and vulnerable groups could be used to address attitudes, misconceptions, and biases that obstruct diversity in research. Continuous quality improvement can be expanded to include granular assessments of equity to analyze quality of healthcare services and uniformity of health outcomes across subgroups. Demand creation and decentralization can increase involvement in and access to clinical trials and high-quality healthcare.

## Implementation outcomes and equity metrics

Implementation outcomes like acceptability, feasibility, appropriateness, adoption, fidelity, and sustainability of interventions—including those promoting equity—are essential to determining whether implementation strategies are effective. It is critical to establish metrics for quantifying and monitoring the degree of engagement with vulnerable populations; for example, the COVID health equity dashboard and hospital racial inclusivity indexing [8,9,13,14]. Implementation outcomes and metrics are instrumental in quantifying the impacts of inequity on healthcare access and ultimately improving health outcomes in vulnerable subgroups.

## Pragmatic research designs

Contrasting with well-established tenets of randomized controlled efficacy trials, pragmatic research designs favor external generalizability by considering both context and effectiveness for real-world implementation interventions. Various designs can be employed including hybrid effectiveness-implementation research and mixed methods designs [15]. Selected elements of these designs can be embedded into clinical research planning to increase inclusivity and diversity. For instance, formative qualitative evaluations to understand the needs of marginalized communities can build trust, rapport, and engagement. Quantifying inclusion, i.e., numbers or proportion of participants from marginalized groups, can foster accountability and tracking. Post-publication, researchers are encouraged to consider mechanisms for expanding and delivering interventions, with special consideration given to those least likely to access them—the highlighted tools offer systematic guides. Lastly, summative qualitative evaluations can be used to understand healthcare practice gaps that hamper successful clinical encounters and access to preventive services and life-saving interventions.

While we cannot address all elements and layers of inequity in health in one swoop, it is important to recognize, understand, and quantitatively monitor them. We need to look at the inputs and outputs of research and healthcare differently and redefine success measures. New imperatives must be set that undergird intentional inclusion of underrepresented—often voiceless—communities as one of the priority markers of success. The tools of IS can be used to ensure that research is designed for dissemination and implementation—which means it is adaptable depending on context and different scenarios. Funders, researchers, journals, policy makers, health system experts, and health providers need to take on this challenge and do the extra work to bridge the gap. IS could, quite literally, put health equity back on the fast track.

## Abbreviations

CFIR: Consolidated Framework for Implementation Research
COVID-19: Coronavirus Disease 2019
IS: implementation science
NCD: noncommunicable disease
NCHS: National Center for Health Statistics
RE-AIM: Reach Effectiveness Adoption Implementation Maintenance
UHC: universal health coverage
